# On the causal structure between CO_2_ and global temperature

**DOI:** 10.1038/srep21691

**Published:** 2016-02-22

**Authors:** Adolf Stips, Diego Macias, Clare Coughlan, Elisa Garcia-Gorriz, X. San Liang

**Affiliations:** 1 European Commission, Joint Research Centre, Institute for Environment and Sustainability, Water Research Unit, Via E. Fermi 2749, Ispra 21027, Italy; 2 School of Marine Sciences, Nanjing Institute of Meteorology, 219 Ningliu Blvd, Nanjing 210044, China

## Abstract

We use a newly developed technique that is based on the information flow concept to investigate the causal structure between the global radiative forcing and the annual global mean surface temperature anomalies (GMTA) since 1850. Our study unambiguously shows one-way causality between the total Greenhouse Gases and GMTA. Specifically, it is confirmed that the former, especially CO_2_, are the main causal drivers of the recent warming. A significant but smaller information flow comes from aerosol direct and indirect forcing, and on short time periods, volcanic forcings. In contrast the causality contribution from natural forcings (solar irradiance and volcanic forcing) to the long term trend is not significant. The spatial explicit analysis reveals that the anthropogenic forcing fingerprint is significantly regionally varying in both hemispheres. On paleoclimate time scales, however, the cause-effect direction is reversed: temperature changes cause subsequent CO_2_/CH_4_ changes.

During the past five decades, the earth has been warming at a rather high rate ((1951–2012; 0.12 [0.08 to 0.14] °C per decade), International Panel of Climate Change, IPCC-2013)^1^, resulting in a scientific and social concern. This warming trend is observed in both field^2^,^3^ and model data^4^, and affects the atmosphere both over the land and over the ocean. Based on the published evidence IPCC attributes this temperature increase to the total increase in radiative forcing and asserts that this is primarily caused by the increase in the atmospheric concentration of CO_2_ during the last 200 years. However, the warming rate changes with time, raising questions regarding the causes underlying the observed trends^5^. Another problem is the relatively large uncertainty in the different external forcing components^6^,^7^,^8^,^9^ that consequently leads to a rather large scatter in the global climate simulations and may have overestimated recent global warming^10^,^11^. The following phrase from the executive summary of Ch 10. of the recent IPPC-2013 assessment (after stating that humans activities extremely likely caused more than half of the observed GMTA increase) might serve for summarising the actual situation: “Uncertainties in forcings and in climate models’ temperature responses to individual forcings, and difficulty in distinguishing the patterns of temperature response due to greenhouse gases and other anthropogenic forcings prevent a more precise quantification of the temperature changes attributable to greenhouse gases”.

Therefore ‘detection’ and ‘attribution’ are still regarded as key priorities in climate change research. IPCC defines ‘detection’ as the process of demonstrating that climate has changed in some statistical sense, which means that the likelihood of occurrence by chance due to internal variability alone is small. Besides statistical analysis of observed data, typically climate models are used to predict the expected responses to external forcing and then the consistency of this response pattern is evaluated with respect to different components of the climate system^1^.

The more challenging problem is to ‘attribute’ this detected climate change to the most likely external causes within some defined level of confidence. As already noted in the Third Assessment Report^11^, unequivocal attribution would require controlled experimentation with the climate system. Since that is not possible, in practice attribution of anthropogenic climate change is understood to mean demonstration that a detected change is ‘consistent with the estimated responses to the given combination of anthropogenic and natural forcing’ and ‘not consistent with alternative, physically plausible explanations of recent climate change that exclude important elements of the given combination of forcings^12^. Therefore attribution analysis is mainly performed through the application of Global Circulation Models that allow testing for causal relationships between anthropogenic forcing, natural variability and temperature evolutions.

Pattern-based fingerprint studies pioneered by^13^ have provided strong scientific evidence of a significant human influence on global atmospheric temperature changes. Recent updates based on multimodel analysis^14^,^15^,^16^ confirm that the primary components of external forcing over the past century are human-caused increases in greenhouse gases, stratospheric ozone depletion and change in atmospheric aerosol content, all reflecting human influence on climate^16^,^17^.

A recent modelling study^18^ confirms CO_2_ as the principal knob governing earth’s temperature. Despite principal plausibility being achieved in this way there are still several open research questions, one being the “missing heat”^10^,^11^,^19^. Also, as the state-of-the-art climate models mostly overestimated the global warming during the last 20 years^10^, additional data driven and model independent corroboration is desirable to support the attribution assessment^1^.

As a common practice in data-based attribution studies, the above-mentioned consistency assessment is usually through correlation and/or regression analysis. A fundamental problem here, however, is that correlation between different variables does not necessarily imply causation^20^. As stated by Barnard^21^: “That correlation is not causation is perhaps the first thing that must be said.” Therefore the actual high correlation between rising CO_2_ levels and increasing surface temperatures alone is insufficient to prove that the increased radiative forcing resulting from the increasing GHG atmospheric concentrations is indeed causing the warming of the earth. Another problem contributing to the remaining uncertainty is the unclear feedback mechanism between global temperature variability and GHG dynamics^22^,^23^ that could contribute to amplify the global warming rate. A first study based on statistical methods for testing causality of human influence on climate^24^ applied Granger causality^25^. They found bi-directional causality between Northern and Southern Hemisphere temperatures, a result that is however not conclusive as these temperatures are not independent from each other and are both driven by the global forcing. Further work^26^,^27^,^28^ based on an improved methodology however confirmed that anthropogenic forcings seem to “Granger cause” temperature changes. In this study, we use a recently developed mathematical method^29^,^30^,^31^,^32^, which is capable of quantitatively evaluating the drive and feedback causal relation between time series, to address the importance of the different forcing components on climate in a quantitative but model independent way. This new method is based on the information flow (IF) concept^31^. The whole new formalism is derived from first principles, rather than as an empirically defined ansatz, with the property of causality guaranteed in proven theorems. This is in contrast to other causality analyses, say that based on Granger causality^25^,^33^,^34^ or convergent cross mapping (CCM)^35^. The resulting formula is concise in form, involving only the common statistics, namely sample covariances. It also allows an explicit discrimination between correlation and causality: causation implies correlation, but not vice versa. For more details, refer to the method section.

## Results and Discussion

We use this technique to analyse the recently measured global mean surface air temperature anomalies (GMTA)^36^ and various reconstructed external forcings covering the period from 1850 to 2005 (156 years)^37^. To introduce the method we calculate the information flow (IF) in nat (natural unit of information) per unit time [nat/ut] from the 156 years annual time series of global CO_2_ concentration to GMTA as 0.348 ± 0.112 nat/ut and −0.006 ± 0.003 nat/ut in the reverse direction. Obviously, the former is significantly different from zero, while the latter, in comparison to the former, is negligible. This result unambiguously shows a one-way causality in the sense that the recent CO_2_ increase is causing the temperature increase, but not the other way around. The results prove to be robust against detrending the data (SI, Table SI2), selecting shorter time periods as e.g. using only the last 100 years, or against using decadal means only (results not shown). It is difficult to achieve a similarly clear result when using Granger causality, as in this case the reverse causality between GMTA and CO_2_ forcing is also significant whereas with CCM only the direction from GMTA to CO_2_ is found to be significant (SI, Tables SI-1 and SI-2).

The atmospheric CO_2_ content serves only as proxy for its radiative forcing and therefore we now examine in more detail the causal relations between the major climate forcings and GMTA. The correlation and the IF between the major reconstructed radiative forcings^37^ (for the used identifiers in^37^ see SI, Table SI-3) and the GMTA time series are given in Table 1, correlations and causations significant at the 95% level and that are larger than 0.1 nat/ut are in bold. The calculated significant IF from the total radiative forcing to GMTA (Table 1) is basically in agreement with results presented by^28^ (their Table 2, Model I), finding a significant one-directional Granger causality between these two variables. The calculated Granger causalities between the different forcing components and GMTA are also largely in agreement with the IF results (SI, Table SI-2). However the non-quantitative nature of Granger causality makes it difficult to disregard the significant reverse causalities from GMTA to anthropogenic and greenhouse gas forcing, as could be done in case of the very small reverse information flows.

The values in Table 1 clearly confirm that the total greenhouse gases (GHG), especially the CO_2_, are the main drivers of the changing global surface air temperature. The radiative forcing caused by aerosols and aerosol-cloud interactions is also important, but significantly smaller (0.2 vs. 0.3 nat/ut). Neither solar irradiance nor volcanic forcing contributes in a significant manner to the long-term GMTA evolution. This is true in spite of short episodes of volcanic forcing that are clearly visible in the time series as they are of insufficient strength to make significant long-term contributions to the GMTA dynamics. Selecting only short data records around a volcanic eruption will however result in a significant causality relation (SI, Table SI-4) for that specific period. For the known major natural modes, the information flows between the Pacific Decadal Oscillation (PDO) and Atlantic Multidecadal Oscillation (AMO) from and to the global surface temperatures are close to 0.0, so essentially no causality relations could be identified here, in contrast to the significant correlation between AMO and GMTA time series (Table 1). This is a good real world example that illustrates the basic fact: correlation does not mean causation. It further questions the assumed fundamental role of the AMO for the global climate as speculated in^38^.

**Figure 1:**
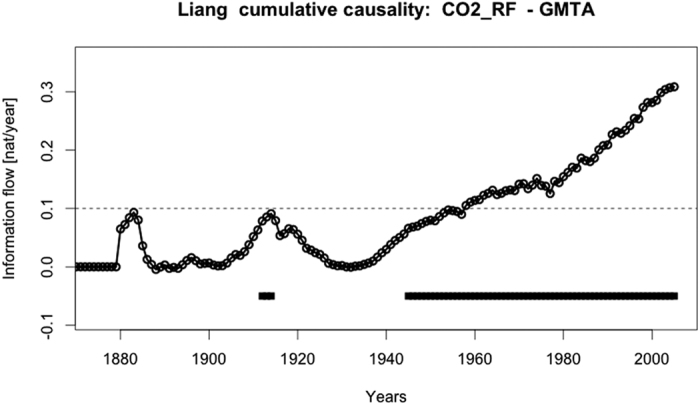
Global information flow from radiative CO_2_ forcing to GMTA. Shown is the time dependence of the information flow between CO_2_ forcing and GMTA when calculating segments with increasing lengths beginning from 1850 to the actual displayed year. Statistically significant values are indicated by the dark squares in the lower part of the figure and the dashed horizontal line at 0.1 [nat/ut] indicates the threshold for relevant flows.

We also try to determine when human activities started to significantly influence the GMTA. Time dependent change in IF from CO_2_ radiative forcing to GMTA since 1880 is presented in Fig. 1. Significant values larger than 0.1 nat/ut are observed only beginning from about 1960. That this is not an effect of the increasing time series length has been tested see SI, Figs SI-1 and SI-2. In this case a qualitative similar result could have been obtained by using Granger causality (SI Fig. SI-3).

The same approach can be applied to investigate the IF between the different forcing components and model derived global surface temperature. As the models are governed by well-known physical equations we might expect even higher causality measures in this case. Indeed the correlation coefficients between the different forcings and the historical CMIP5 overall ensemble temperatures are all higher compared to the one between forcings and observed GMTA (Table 2). The IF from the total forcing to the simulated GMTA is indeed slightly increased, confirming the important influence of the external forcing on the GMTA. As expected, ensemble averaging has the effect of enhancing the relative importance of the external forcing component compared to internal model variability. However the IF from single forcing components to the simulated GMTA is always significantly smaller and in the case of aerosol forcing and aerosol-cloud interactions even becomes insignificant (Table 2) compared to the IF to the observed GMTA. As this result is in disagreement to the analysis based on observed temperatures it might point to differences with relation to the model specific implementations of aerosol and aerosol-cloud interactions in the CMIP5 models^9^,^39^.

Further we apply this technique to analyse paleoclimatological air temperature (PAT)^40^ and CO_2_/CH_4_ data from the EPICA Dome C ice cores^41^,^42^ from the last 800,000 years. Both time series are interpolated on the same time steps of 1000 years using the AICC2012^43^,^44^ chronology. As already known the two data set are highly correlated with a correlation coefficient of 0.842 ± 0. By calculating the IF in nat per unit time from the 1000 year interpolated PAT time series to CO_2_ concentration we get 0.123 ± 0.060 nat/ut and −0.054 ± 0.040 nat/ut in the reverse direction. Therefore we have on these long time scales a significant IF only from the temperature data to the CO_2_, but not in the other direction, exactly opposite to that seen in the data from the last 156 years. This result proves robust against using different ice age/gas age chronologies (SI, Tables SI-5 and SI-6 comparing EDC3 and AICC2012 chronology) and against using the recent corrected CO_2_ data from Bereiter^45^ (SI, Table SI-7). The time step chosen for interpolation influences neither the strong correlation (always around 0.88 for the EDC3 chronology) nor the significant causation (SI, Table SI-8). This supports the hypothesis that on geological time scales air temperature changes are causing the subsequent changes in CO_2_ concentration. This was already hypothesized by^46^, who claimed that CO_2_ lagged Antarctic deglacial warming by 800 ± 200 years, during a specific deglaciation event (Termination III ~ 240,000 years ago). Recently Parrenin *et al.*^47^, did not find any significant asynchrony in the timing between atmospheric CO_2_ and Antarctic temperature changes during the last deglaciation event (Termination TI). If we apply causality analysis only to data from event TI (22000–10000 years), we do get a bidirectional significant flow of 0.120 ± 0.074 nat/ut from PAT to CO_2_ and 0.484 ± 0.168 nat/ut from CO_2_ to PAT pointing to a synchronous behaviour or even a leading CO_2_ signal (see Table SI-9). Using the old EDC3 chronology would have given a very different result, with CO_2_ changes clearly causing PAT changes (Table SI-10). Because of the inherent nonlinear dynamics of the climate system, changes in correlation during single events could even be expected^35^. The causality analysis indicates that for the full 800,000 years time series PAT is indeed leading CO_2_ because of the significant IF from PAT to CO_2_. This is in principal agreement with the conclusion from Nes *et al.*^48^ that has been derived using convergent cross mapping. However, when interpolating to time steps longer than 3000 years the IF decreases (Table SI-8). Because of this it is not possible to specify a time lag of maximal IF in contrast to the 6000 year time lag found by Nes *et al.*^48^. Data from another strong greenhouse gas, namely methane CH_4_, are also available from EPICA Dome C covering the same time period as the CO_2_ data^49^. Again, as for CO_2_, we find a strong significant correlation between PAT and CH_4_ of 0.777. The IF from PAT to CH_4_ for the interpolated time series (1000 years time step) equals 0.393 ± 0.051 nat/ut and 0.007 ± 0.025 nat/ut in the reverse direction. Therefore the causal drive of temperature on the CH_4_ dynamics is even stronger than for CO_2_. This supports the expectation that on paleoclimatological time scales changing temperature could be held responsible for following changes in greenhouse gas (CO_2_/CH_4_) concentrations.

**Figure 2:**
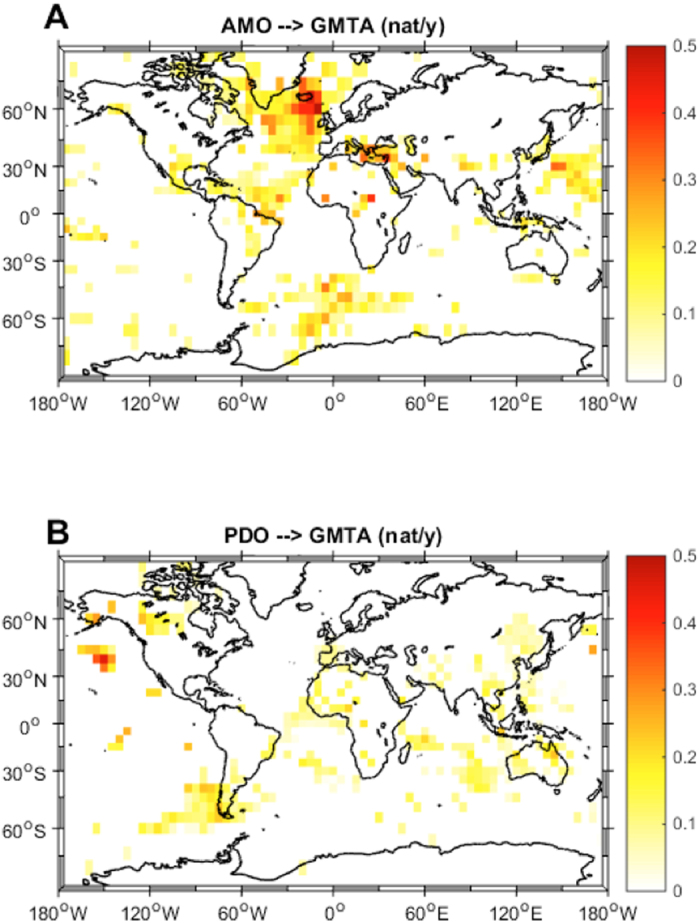
Global information flow from internal climate variability to GMTA. Shown is the spatial distribution of the information flow between the Atlantic Multidecadal Oscillation (AMO) and the gridded global mean temperature anomalies (GMTA) (**A**) and the distribution of the information flow between the Pacific Decadal Oscillation (PDO) and the gridded global mean temperature anomalies (GMTA) (**B**). The maps were created by the authors using the m-map toolbox included in Matlab®.

**Figure 3:**
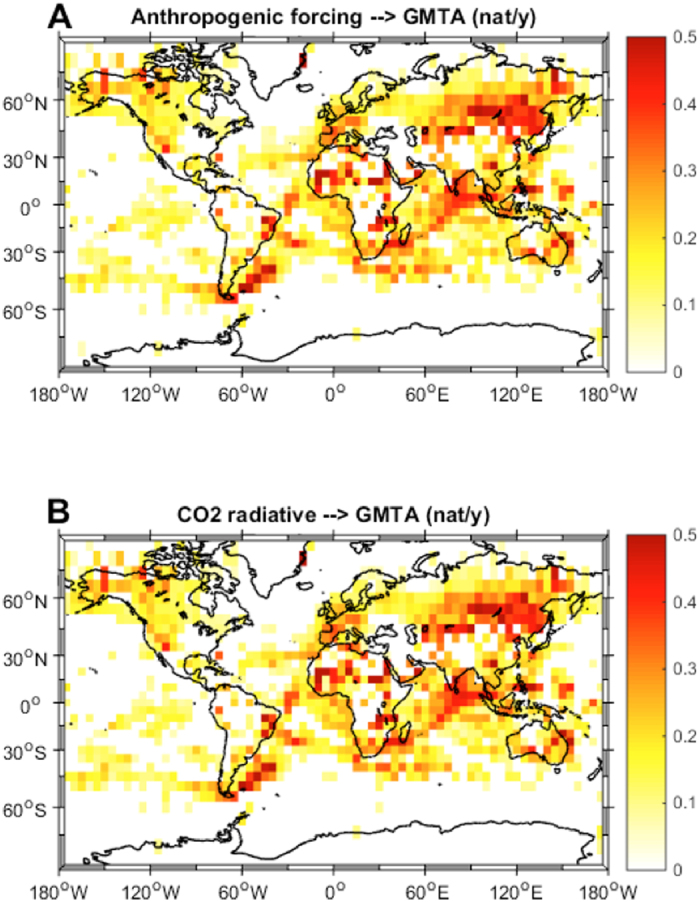
Global information flow from anthropogenic forcing to GMTA. Shown is the spatial distribution of the information flow between the total anthropogenic forcing and the gridded global mean temperature anomalies (GMTA) (**A**) and the spatial distribution of the information flow between the radiative forcing caused by CO2 and the gridded global mean temperature anomalies (GMTA) (**B**). The maps were created by the authors using the m-map toolbox included in Matlab®.

**Figure 4:**
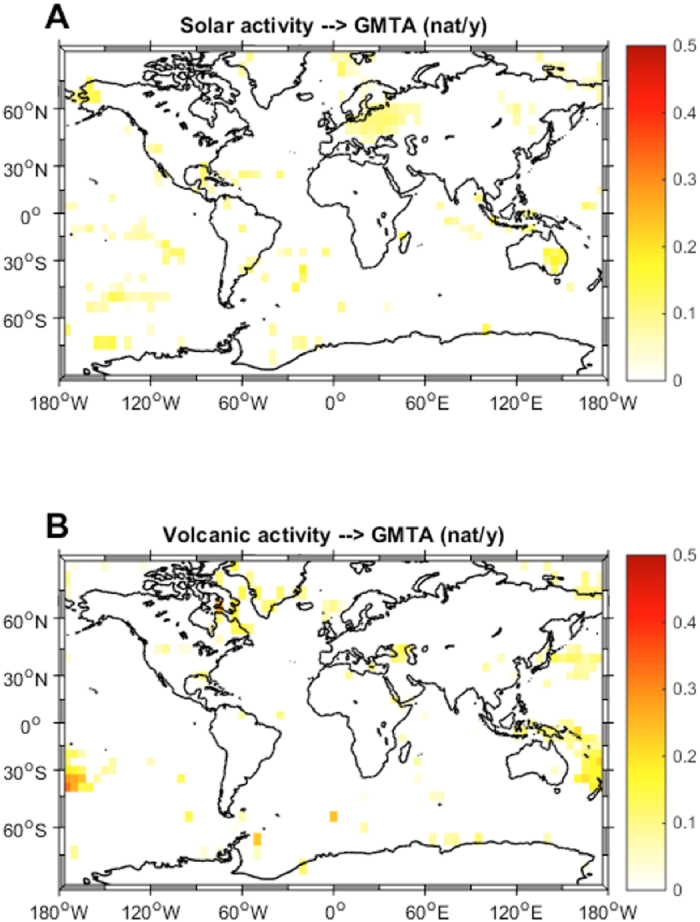
Global information flow from natural forcing to GMTA. Shown is the spatial distribution of the information flow between solar forcing and the gridded global mean temperature anomalies (GMTA) (**A**) and the spatial distribution of the information flow between the radiative forcing caused by volcanic activity and the gridded global mean temperature anomalies (GMTA) (**B**). The maps were created by the authors using the m-map toolbox included in Matlab®.

Another crucial research question that we can address with this new method is “where is the recently increasing anthropogenic forcing likely to cause the most pronounced consequences?”. In order to assess which regions of the earth are more ‘sensitive’ to anthropogenic forcings and where natural modes of variability contribute more to the temperature series, we applied the same type of causality analysis as used in^29^ to the globally-gridded GMTA product. Due to the historically sparse data availability we could use only data from 1950 onwards and excluded regions South and North of 70 degrees. Looking first at the natural climate modes we see that PDO mainly shows significant IF over the North Pacific (Fig. 2A) and AMO mainly over the North Atlantic (Fig. 2B). This kind of expected result provides an additional first order validation of the method when applied to climate data. When analysing the IF from the global anthropogenic forcing to the GMTA (Fig. 3), in the Northern Hemisphere, we identified several regions of significant high causality. For example, IF takes largest values in Europe, North America, and China, densely populated and industrialized areas having shown strong recent warming^2^. On the other hand there are also regions with high causality like Siberia, the Sahel zone and Alaska that are not that much influenced by human activities. In the Southern Hemisphere, however, this IF distribution displays a most unexpected pattern, with high values in a large swath of the southern Atlantic, South Africa, parts of the Indian Ocean and Australia. This is true for both the total anthropogenic forcing (Fig. 3A) and the radiative forcing caused by CO_2_ alone (Fig. 3B). Therefore, despite CO_2_ being a globally well-mixed gas, the IF to surface temperature is regionally very different, showing sensitive areas. Indeed, most of these depicted sensitive regions have shown especially strong warming during the last 60 years see Fig. 4 of^2^. Analysis of the spatial distributions of the IFs between solar forcing and GMTA (Fig. 4A) and volcanic forcing and GMTA (Fig. 4B) shows that over the considered period these flows are basically insignificant, in agreement with the previous analysis with the global mean values.

## Conclusions

Using the IF concept we were able to confirm the inherent one-way causality between human activities and global warming, as during the last 150 years the increasing anthropogenic radiative forcing is driving the increasing global temperature, a result that cannot be inferred from traditional time delayed correlation or ordinary least square regression analysis. Natural forcing (solar forcing and volcanic activities) contributes only marginally to the global temperature dynamics during the last 150 years. Human influence, especially via CO_2_ radiative forcing, has been detected to be significant since about the 1960s. This provides an independent statistical confirmation of the results from process based modelling studies. Investigation of the temperature simulations from the CMIP5 ensemble is largely in agreement with the conclusion drawn from the observational data. However on very long time scales (800,000 years) the IF is only significant in the direction from air temperature to CO_2_. This supports the idea that the feedback of GHGs to temperature changes seems to be much slower than the fast response of temperature to changes in GHGs^48^.

The spatial explicit analysis strongly indicates that the increasing anthropogenic forcing is causing very differing effects regionally with some regions in the southern hemisphere showing large IF values. Regions of significant IF do coincide with regions having stronger than average recent warming trends. Our observational data-based study, therefore, not only provides complementary support for the results from global circulation modelling, but also calls for attention for further research in regions of increased sensitivity to the forcing resulting from anthropogenic activities.

## Material and Methods

The global mean surface air temperature anomalies were obtained from the HadCRUT4 dataset^36^,^50^. Datasets spanning the period 1850–2013 were obtained for the global mean temperature, temperatures of the Southern and Northern Hemispheres; the gridded data have a 5° × 5° resolution. The Meinshausen historical forcing data^37^,^51^ cover the period from 1765 to 2005. The overlap period of the two datasets, 1850–2005 (156 years), is hence chosen for our analysis. Air temperature, CO_2_ and CH_4_ data for the last 800,000 years from the Antarctic EPICA Dome C ice cores^40^,^41^,^42^,^49^ were obtained from^51^. These non-equidistant temperature, CO_2_ and CH_4_ data were interpolated on different equidistant time steps using Akima^52^ spline interpolation. The ice core data were downloaded from the NOAA ice core data base (ftp://ftp.ncdc.noaa.gov/pub/data/paleo/icecore/antarctica/ assessed last time October 2015). All CMIP5 model data were downloaded using the KNMI Climate Explorer^53^.

We follow Liang^29^,^54^ to evaluate the cause-effect relation between time series. Causality is measured as the time rate of information flowing from one time series to another. It has long been recognized that a nonzero IF, or information transfer as it may appear in the literature, from an event to another logically tells the strength of the causality from the former to the latter, and a vanishing causality must entail a zero flow. This recognition is further supported by the recent discovery^34^ that transfer entropy^33^ and Granger causality^25^, the two most extensively studied formalisms of IF and causality analysis respectively, turn out to be equivalent up to a factor of 2.

In causality analysis, a principle (actually the only quantitatively stated fact) that must be verified is that, when the evolution of a dynamical event (say *A*) is independent of another (say *B*), then the causality from *B* to *A* is nil. It has long been found that Granger causality and transfer entropy fail to verify this principle in many applications, giving rise to spurious causalities; for a systematic investigation, see^34^. A remarkable example is examined in^55^, where for a carefully designed chaotic system, transfer entropy (and hence Granger causality test) not only fails to reproduce the preset one-way causality, but, as the parameter varies, the thus-obtained causality can be completely reversed! These problems have been extensively studied in the past decade, and, for a dynamical system, the dependence of transfer entropy and/or Granger causality on the autodependency coefficient has been blamed for the failure (see references in^29^,^54^).

One may argue that IF or causality is a real physical notion, and a real physical notion should be rigorously formulated, rather than empirically proposed as what have been done before. Indeed, recently it has been shown that IF actually can be derived from first principles. Given a two-dimensional system


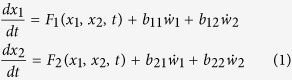


where 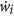 (*i* = 1, 2) indicate white noises; 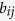 and 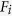 are arbitrary functions of *X* and *t*, Liang^35^ proved that the rate of information (in terms of Shannon entropy) flowing from 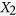 to 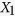 is:


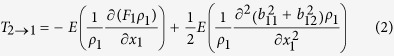


where 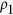 is the marginal density of 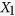, and *E* stands for mathematical expectation (units: nats per unit time). Remarkably, Eq. (2) has the above stringent principle of causality naturally embedded. This is stated in two theorems in Liang^32^, which read, loosely speaking, if the evolution of 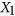 is independent of 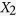, then 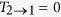.

When only two time series, say 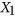 and 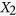, are given, we first need a model to fulfill the IF evaluation. For linear systems, the maximum likelihood estimator of (2) turns out to be very concise in form^29^:


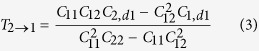


where 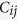 (*i, j* = *1,* 2) is the sample covariance between 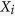 and 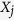, and 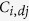 the sample covariance between 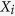 and 
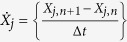
, with Δ*t* being the time stepsize (units nats per unit time). If 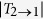 is nonzero, 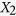 is causal to 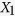; if not, it is noncausal.

The above tight formula is explicitly expressed in terms of the sample covariances of the involved time series and their derivatives. In a strict sense, it is precise only for linear systems (the original Eq. (2) applies to any systems, though), but the validations have shown that it is a good approximation for nonlinear time series, and has seen remarkable success with highly nonlinear touchstone systems, such as the aforementioned system in^55^ which fails transfer entropy and hence Granger causality tests. The formula also confirms in a quantitative way that causation implies correlation, whereas correlation does not imply causation. The quantitative nature of this formulation allows disregarding small absolute information flows (<0.1 nat/ut) as insignificant as is done in this article.

The confidence interval estimation also follows Liang^29^,^54^; it is based on the observation that, for a large ensemble, the maximum likelihood estimate of a parameter approximately obeys a normal distribution around its true value. All given confidence intervals are significant at the 95% level. Analysis software was coded in R and MATLAB (for generating the maps). Using the respective implementations of Granger causality and CCM in the R software packages “MSBVAR”, “lmtest” and “multispatialCCM” we could perform comparisons with Liang causality^56^.

## Additional Information

**How to cite this article**: Stips, A. *et al.* On the causal structure between CO_2_ and global temperature. *Sci. Rep.* **6**, 21691; doi: 10.1038/srep21691 (2016).

## Supplementary Material

Supporting Information

## Figures and Tables

**Table 1:** Correlation and information flow between observed global surface temperature and different external forcing’s and internal climate variations.

Radiative Forcing	Correlation and Causality–HADCRUT4		
	*Correlation*	*Forcing*→*GMTA*[nat/year]	*GMTA*→*Forcing*[nat/year]
Total forcing	0.804 ± 0	0.244 ± 0.091	0.036 ± 0.080
Anthropogenic	0.863 ± 0	0.355 ± 0.112	−0.008 ± 0.005
All GHG	0.852 ± 0	0.318 ± 0.108	−0.005 ± 0.003
CO2	0.852 ± 0	0.316 ± 0.108	−0.003 ± 0.003
Aerosol	−0.810 ± 0	0.232 ± 0.095	−0.002 ± 0.006
Cloud	−0.796 ± 0	0.208 ± 0.092	−0.001 ± 0.004
Solar	0.616 ± 0	0.082 ± 0.059	0.035 ± 0.051
Volcanic	0.089 ± 0.267	0.003 ± 0.006	−0.004 ± 0.009
AMO (1900–2008)	0.477 ± 0	0.018 ± 0.043	0.021 ± 0.014
PDO (1900–2008)	0.123 ± 0.204	−0.002 ± 0.013	−0.011 ± 0.025

The chosen unit time step is ut = 1 year.

**Table 2:** Correlation and information flow between global surface temperature and different CMIP5 forcing’s and internal climate variations.

Radiative Forcing	Correlation and Causality – CMIP5		
	*Correlation*	*Forcing*→*GMTA*[nat/year]	*GMTA*→*Forcing*[nat/year]
Total forcing	0.934 ± 0	0.253 ± 0.077	0.010 ± 0.164
Anthropogenic	0.929 ± 0	0.159 ± 0.079	−0.014 ± 0.008
All GHG	0.918 ± 0	0.133 ± 0.074	−0.009 ± 0.004
CO2	0.923 ± 0	0.140 ± 0.076	−0.005 ± 0.004
Aerosol	−0.868 ± 0	0.074 ± 0.058	−0.004 ± 0.009
Cloud	−0.865 ± 0	0.069 ± 0.057	−0.005 ± 0.006
Solar	0.667 ± 0	−0.004 ± 0.032	0.063 ± 0.051
Volcanic	0.216 ± 0.009	0.006 ± 0.007	−0.015 ± 0.023
AMO (1900–2005)	0.324 ± 0.001	−0.005 ± 0.013	0.018 ± 0.008
PDO (1900–2005)	0.098 ± 0.316	0.003 ± 0.004	−0.004 ± 0.004

The chosen unit time step is ut = 1 year.
