# Supplementary material for: On the causal structure between CO2 and global temperature
Source: Sci Rep. 2016 Feb 22;6:21691. doi: 10.1038/srep21691 (PMC4761980; doi:10.1038/srep21691)

Supporting Information:

On the causal structure between CO_2_ and global temperature

*Authors:* Adolf Stips^1*^, Diego Macias^1^, Clare Coughlan^1^, Elisa Garcia-Gorriz^1^, X. San Liang^2^

**Table SI–1**

Causality between CO_2_ and GMTA time series (1850-2012) based on different causality measures. We find significant information flow from CO2 to GMTA, significant bi-directional causation using Granger causality and significant causation directed from GMTA to CO2 using CCM method. This reveals quite clearly the problem of not detecting *nil causality* with the other methods.

|  | **Causality measures: CO_2_ – HADCRUT4** | | | |
| --- | --- | --- | --- | --- |
|  | ***CO2 →*** ***GMTA*** | | ***GMTA→ CO2*** | |
|  | **[nat/ut]** | **95%** | **[nat/ut]** | **95%** |
| Information flow | **0.348** | **±0.112** | -0.006 | ±0.003 |
|  | **F** | **p** | **F** | **p** |
| Granger | **36.23** | **1.171e-08** | **10.81** | **1.244e-03** |
| CCM |  | 0.08 |  | **0** |

**Table SI–2**

Causality between detrended CO_2_ and detrended GMTA time series (1850-2012) based on different causality measures. We find significant information flow from CO2 to GMTA, also significant directional causation using Granger causality and significant causation directed from GMTA to CO2 using CCM method. This reveals quite clearly the problem of not detecting *nil causality* with CCM method.

| **Detrended data** | **Causality measures: CO_2_ – HADCRUT4** | | | |
| --- | --- | --- | --- | --- |
|  | ***CO2 →*** ***GMTA*** | | ***GMTA→ CO2*** | |
|  | **[nat/ut]** | **95%** | **[nat/ut]** | **95%** |
| Information flow | **0.162** | **±0.077** | -0.006 | ±0.007 |
|  | **F** | **p** | **F** | **p** |
| Granger | **16.403** | **7.98e-05** | 2.462 | 0.119 |
| CCM |  | 0.27 |  | **0.01** |

**Table SI-3**

Calculated F-statistics and p-value (significant when <0.05) for Granger causality between different forcings and HADCRUT4 global temperature data. Bold values are significant at the 95% level (p-value < 0.05). Several significant reverse flows reveal quite clearly the problem of not detecting *nil causality* using Granger causality. Further we provide the name of the forcing components as used by Meinshausen et al. 2011 and as used in the carbon cycle model MAGICC6.

| **Radiative Forcing**  **(Abbreviation)** | **Radiative Forcing**  **(MAGICC6 name)** | **Granger Causality: Forcing – HADCRUT4** | | | |
| --- | --- | --- | --- | --- | --- |
|  |  | ***Forcing***→***GMTA*** | | ***GMTA***→***Forcing*** | |
| Value | Value | **F** | **p** | **F** | **p** |
| Total forcing | TOTAL_INCLVOLCANIC_RF | **27.411** | **5.386e-07** | 0.766 | 0.383 |
| Anthropogenic | TOTAL_ANTHRO_RF | **38.006** | **6.065e-09** | **10.414** | **1.532e-03** |
| All GHG | GHG_RF | **32.806** | **5.286e-08** | **14.117** | **2.442e-04** |
| CO2 | CO2_RF | **32.480** | **6.069e-08** | **5.656** | **1.863e-02** |
| Aerosol | TOTAER_DIR_RF | **22.321** | **5.207e-06** | 0.502 | 0.479 |
| Cloud | CLOUD_TOT_RF | **19.495** | **1.904e-05** | 0.340 | 0.560 |
| Solar | SOLAR_RF | **7.149** | **0.008** | 1.770 | 0.185 |
| Volcanic | VOLCANIC_ANNUAL_RF | 0.640 | 0.425 | 0.9579 | 0.329 |

**Table SI-4**

Correlation and causality [nat/ut] between forcing caused by short volcano eruptions and the GMTA time series using selected time periods with and without volcanic eruptions.

| **Time Period : Volcanic Radiative Forcing** | **Liang Causality: Volcanic Forcing – HADCRUT4** | | |
| --- | --- | --- | --- |
|  | ***Correlation*** | ***Forcing***→GMTA | GMTA→***Forcing*** |
| 1850-2005 | 0.090±0.267 | 0.003±0.006 | -0.004±0.009 |
| 1880-1890 (Krakatoa) | **0.618±0.044** | **0.600±0.447** | -0.261±0.390 |
| 1960-1970 (Agung) | **0.547±0.082** | **0.443±0.284** | -0.224±0.276 |
| 1990-2000 (Pinatubo) | **0.652±0.030** | **0.479±0.409** | -0.091±0.426 |
| 1950-1960(No Eruption) | 0.015±0.965 | 0.010±0.021 | -0.19±0.026 |
| 1960-2005 | **0.301±0.042** | 0.037±0.052 | -0.006±0.064 |

**Table SI-5**

Correlation and information flow between EPICA CO_2_/CH_4_ and paleoclimatological air temperature (800 ky, all based on EDC3 chronology). The unit time step chosen is ut=1000 years.

| EDC3 time scale | **Causality measures: CO_2_ – Temperature** | | | |
| --- | --- | --- | --- | --- |
|  | ***CO2 → PAT*** | | ***PAT→ CO2*** | |
| Correlation | **0.880** | **±0** |  |  |
|  | **[nat/ut]** | **95%** | **[nat/ut]** | **95%** |
| Information flow | -0.002 | ±0.040 | **0.166** | **±0.038** |
|  | **F** | **p** | **F** | **p** |
| Granger causality | 0.012 | 0.913 | **72.544** | **0** |
| EDC3 time scale | **Causality measures: CH_4_ – Temperature** | | | |
|  | ***CH4 → PAT*** | | ***PAT→ CH4*** | |
| Correlation | **0.820** | **±0** |  |  |
|  | **[nat/ut]** | **95%** | **[nat/ut]** | **95%** |
| Information flow | -0.040 | ±0.030 | **0.335** | **±0.048** |
|  | **F** | **p** | **F** | **p** |
| Granger causality | **6.965** | **0.008** | **188.164** | **0** |

**Table SI-6**

Correlation and information flow between EPICA CO_2_/CH_4_ and paleoclimatological air temperature (800ky, all based on AICC2012 chronology). The unit time step chosen is ut=1000 years.

| AICC2012 time scale | **Causality measures: CO_2_ – Temperature** | | | |
| --- | --- | --- | --- | --- |
|  | ***CO2 → PAT*** | | ***PAT→ CO2*** | |
| Correlation | **0.842** | **±0** |  |  |
|  | **[nat/ut]** | **95%** | **[nat/ut]** | **95%** |
| Information flow | 0.054 | ±0.039 | **0.123** | **±0.060** |
|  | **F** | **p** | **F** | **p** |
| Granger causality | **7.724** | **0.006** | **15.739** | **0** |
| AICC2012 time scale | **Causality measures: CH_4_ – Temperature** | | | |
|  | ***CH4 → PAT*** | | ***PAT→ CH4*** | |
| Correlation | **0.777** | **±0** |  |  |
|  | **[nat/ut]** | **95%** | **[nat/ut]** | **95%** |
| Information flow | -0.007 | ±0.025 | **0.393** | **±0.051** |
|  | **F** | **p** | **F** | **P** |
| Granger causality | 0.268 | 0.605 | **228.868** | **0** |

**Table SI-7**

Correlation and information flow between EPICA CO_2_ and paleoclimatological air temperature (800ky, all based on AICC2012 chronology, but corrected data according to Bereiter et al. 2015). The unit time step chosen is ut=1000 years.

| AICC2012 time scale  (Bereiter et al. 2015) | **Causality measures: CO_2_ – Temperature** | | | |
| --- | --- | --- | --- | --- |
|  | ***CO2 → PAT*** | | ***PAT→ CO2*** | |
| Correlation | **0.905** | **±0** |  |  |
|  | **[nat/ut]** | **95%** | **[nat/ut]** | **95%** |
| Information flow | -0.079 | ±0.043 | **0.135** | **±0.040** |
|  | **F** | **p** | **F** | **P** |
| Granger causality | **12.72** | **0.001** | **43.58** | **0** |

**Table SI-8.** Correlation and information flow between EPICA CO_2_ and paleoclimatological air temperature (based on EDC3 chronology) for different interpolation time steps ∆t. The unit time step chosen here is ut=1000 years.

| Time step ∆t [years] | Correlation | CO2 → Temp [nats/ut] | Temp → CO2 [nats/ut] |
| --- | --- | --- | --- |
| 500 | **0.880**±0 | 0.046±0.042 | **0.159**±0.036 |
| 750 | **0.879**±0 | 0.019±0.040 | **0.176**±0.037 |
| 1000 | **0.880**±0 | -0.002±0.040 | **0.166**±0.038 |
| 1250 | **0.883**±0 | -0.006±0.039 | **0.156**±0.039 |
| 1500 | **0.880**±0 | -0.012±0.040 | **0.165**±0.040 |
| 1750 | **0.880**±0 | -0.001±0.041 | **0.133**±0.040 |
| 2000 | **0.876**±0 | -0.026±0.041 | **0.150**±0.041 |
| 3000 | **0.880**±0 | -0.041±0.040 | **0.141**±0.042 |
| 4000 | **0.862**±0 | -0.016±0.037 | 0.092±0.037 |
| 5000 | **0.880**±0 | -0.010±0.041 | 0.054±0.040 |

**Table SI-9**

Correlation and information flow between EPICA CO_2_ and paleoclimatological air temperature (based on AICC2012 chronology) for termination I (Parrenin et al. 2013). The unit time step chosen is ut=100 years.

| Termination I  22000-10000  AICC2012 time scale | **Causality measures: CO_2_ – Temperature** | | | |
| --- | --- | --- | --- | --- |
|  | ***CO2 → PAT*** | | ***PAT→ CO2*** | |
| Correlation | **0.970** | **±0** |  |  |
|  | **[nat/ut]** | **95%** | **[nat/ut]** | **95%** |
| Information flow | **0.484** | **±0.168** | **0.120** | **±0.074** |
|  | **F** | **p** | **F** | **p** |
| Granger causality | **9.967** | **0.002** | **31.164** | **0** |

**Table SI-10**

Correlation and information flow between EPICA CO_2_ and paleoclimatological air temperature (based on EDC3 chronology) for termination I (Parrenin et al. 2013). The unit time step chosen is ut=100 years.

| Termination I  22000-10000  EDC3 time scale | **Causality measures: CO_2_ – Temperature** | | | |
| --- | --- | --- | --- | --- |
|  | ***CO2 → PAT*** | | ***PAT→ CO2*** | |
| Correlation | **0.980** | **±0** |  |  |
|  | **[nat/ut]** | **95%** | **[nat/ut]** | **95%** |
| Information flow | **0.727** | **±0.182** | 0.043 | ±0.047 |
|  | **F** | **p** | **F** | **p** |
| Granger causality | **59.720** | **0** | 3.096 | 0.081 |

**Fig. SI-1**

Comparison between cumulative Liang causality (information flow) and Granger causality calculated between total anthropogenic forcing and GMTA time series. In the case of Granger causality the F-statistics is plotted and scaled to the same range as the information flow. The thick lines at the bottom of the figure depict time periods with significant causality at the 95% level. In the case of existing causality Granger causality might therefore provide similar results as with the information flow.


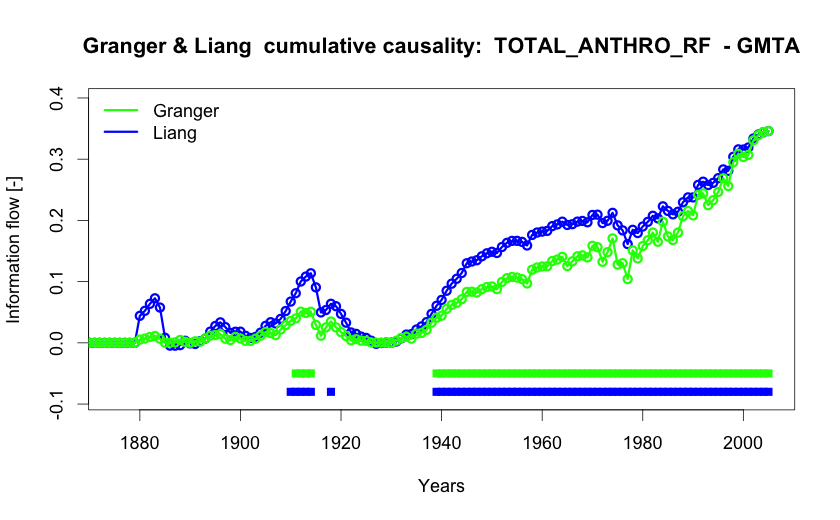


The effect of increasing time-series length on the information analysis was tested using artificially created dependent time-series X and Y (where X cause Y by a linear combination). The information analysis results (Fig. SI-2) do reveal a rapid convergence of the information flow (~1) since the beginning of the cumulative analysis. This demonstrates that the length of the considered time-series did not fundamentally influence the information flow calculations, which are, nonetheless, expressed as a function of the time-step as described above.

**Fig. SI-2**

Liang cumulative information flow for a time series X1 (length 200 years) that is linearly causing X2. White noise is added to both time series. Black squares indicate significant information flow. Immediately when beginning the first calculation the information flow is significant and stays about 1 [nat/year].


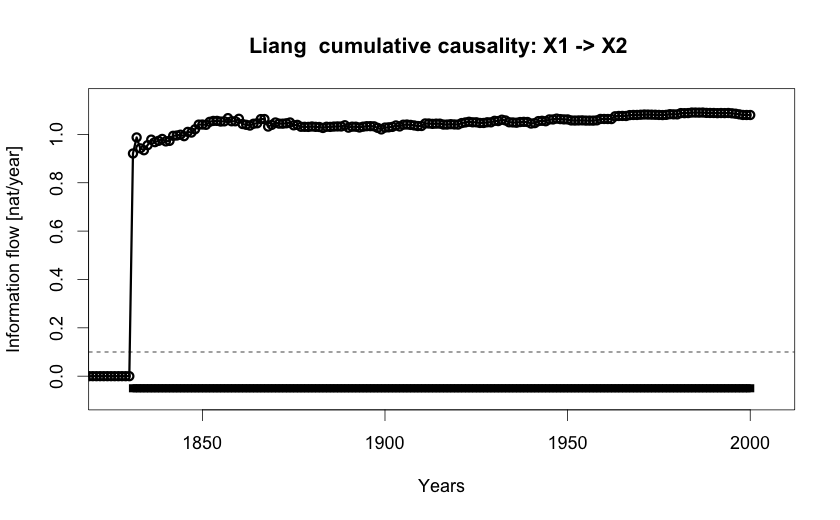


**Fig. SI-3**

Liang cumulative information flow for a time series X1 (length 200) that is linearly causing X2. White noise is added to both time series. However the first 100 elements are replaced by white noise only, so that the signal starts now at element 101. The white noise does not produce any significant information flow. Shortly after the signal starts the information flow is significant and is initially increasing fast before approaching a seemingly stationary behavior. It can be noted that already after a period of only 5 causal related years the calculated information flow is significant, despite the preceding 100 years of white noise.


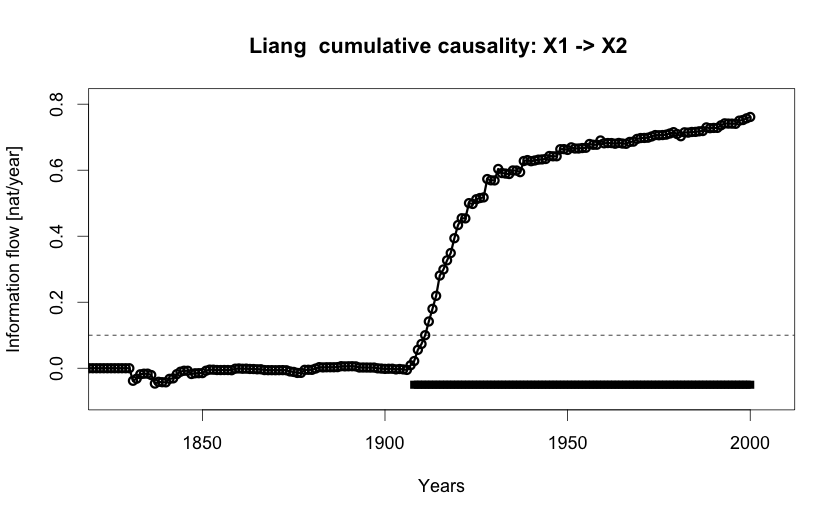

Supplement: Supporting Information [file srep21691-s1.doc]
